# Latency, sleep hours, and blood pressure in hospitalized patients in Mexico

**DOI:** 10.15649/cuidarte.4004

**Published:** 2024-12-19

**Authors:** María Elena Pérez-Vega, Ma. Elena Aguirre-González, Tania Amaro-Valdez, Edgar Noé Morelos-García, Eunice Reséndiz-González, Ricardo Lara-Pérez

**Affiliations:** 1 Universidad Autónoma de Tamaulipas, Tampico Faculty of Nursing. Tamaulipas, México. meperez@docentes.uat.edu.mx Universidad Autónoma de Tamaulipas, Tampico Faculty of Nursing Universidad Autónoma de Tamaulipas Tamaulipas México meperez@docentes.uat.edu.mx; 2 Universidad Autónoma de Tamaulipas, Tampico Faculty of Nursing. Tamaulipas, México. meaguirreg@docentes.uat.edu.mx Universidad Autónoma de Tamaulipas, Tampico Faculty of Nursing Universidad Autónoma de Tamaulipas Tamaulipas México meaguirreg@docentes.uat.edu.mx; 3 Universidad Autónoma de Tamaulipas, Tampico Faculty of Nursing. Tamaulipas, México. tamaro@uat.edu.mx Universidad Autónoma de Tamaulipas, Tampico Faculty of Nursing Universidad Autónoma de Tamaulipas Tamaulipas México tamaro@uat.edu.mx; 4 Universidad Autónoma de Tamaulipas, Tampico Faculty of Nursing. Tamaulipas, México. emorelos@docentes.uat.edu.mx Universidad Autónoma de Tamaulipas, Tampico Faculty of Nursing Universidad Autónoma de Tamaulipas Tamaulipas México emorelos@docentes.uat.edu.mx; 5 Universidad Autónoma de Tamaulipas, Tampico Faculty of Nursing. Tamaulipas, México. eresendi@docentes.uat.edu.mx Universidad Autónoma de Tamaulipas, Tampico Faculty of Nursing Universidad Autónoma de Tamaulipas Tamaulipas México eresendi@docentes.uat.edu.mx; 6 Universidad Autónoma de Tamaulipas, Tampico Faculty of Nursing. Tamaulipas, México. drlpr@hotmail.com Universidad Autónoma de Tamaulipas, Tampico Faculty of Nursing Universidad Autónoma de Tamaulipas Tamaulipas México drlpr@hotmail.com

**Keywords:** Sleep Duration, Sleep Latency, Arterial Pressure, Hypertension, Hospitalization, Duración del Sueño, Latencia del Sueño, Presión Arterial, Hipertensión, Hospitalización, Duração do Sono, Latência do Sono, Pressão Arterial, Hipertensão, Hospitalização

## Abstract

**Introduction:**

Multiple studies have associated sleep duration and quality with changes in blood pressure in the general population, considering it a related risk factor. However, there is limited information regarding hospitalized patients who tend to experience sleep disturbances.

**Objective:**

o examine the relationship between subjective sleep duration, sleep latency, and systolic and diastolic blood pressure in hospitalized patients.

**Materials and Methods:**

A cross-sectional study included 381 Mexican adults hospitalized in a secondary-level hospital in Tampico, Tamaulipas, Mexico. Self-reported sleep duration and latency were assessed using the Pittsburgh Sleep Quality Index component. Clinical and sociodemographic data were collected. The study was approved by an ethics committee.

**Results:**

In adjusted models, a significant negative association was found between subjective sleep hours and diastolic blood pressure (β= -0.59; 95% CI: -1.80 to -0.10). A significant positive relationship was observed between sleep latency and systolic blood pressure (β= 1.48; 95% CI: -0.49 to 2.47).

**Discussion:**

The findings are consistent with those of previous studies. Hospitalization can impact sleep due to unfamiliar environments, noise, irregular schedules, and invasive procedures, affecting patients’ health and prognosis.

**Conclusion:**

It is essential to consider sleep as a modifiable factor in preventing and managing hypertension in hospitalized patients and to find effective interventions to improve sleep duration and quality.

## Resumen

**Introducción:**

Múltiples estudios han asociado la duración y calidad del sueño con cambios en la tensión arterial en la población general, considerándolo un factor de riesgo asociado, pero hay escasa información sobre pacientes hospitalizados, quienes tienden a experimentar alteraciones en el sueño.

**Objetivo:**

Examinar la relación entre la duración subjetiva del sueño, la latencia del sueño y la presión arterial sistólica y diastólica en pacientes hospitalizados.

**Materiales y métodos:**

Estudio transversal de 381 adultos mexicanos hospitalizados en un hospital de segundo nivel en Tampico, Tamaulipas, México. Se evaluó por autorreporte la duración y la latencia de sueño usando el componente del Índice de Calidad del Sueño de Pittsburg. Se obtuvieron datos clínicos y sociodemográficos. Fue aprobado por comité de ética.

**Resultados:**

En los modelos ajustados, se encontró una asociación significativa y negativa entre las horas subjetivas de sueño y la presión arterial diastólica (β= -0,59; IC 95%: -1,80 - -0,10). Se observó una relación significativa y positiva entre la latencia del sueño y la presión arterial sistólica (β= 1,48; IC 95%: -0,49 – 2,47).

**Discusión:**

Los resultados son similares a los de estudios previos. La hospitalización puede afectar el sueño debido a entornos desconocidos, ruidos, horarios irregulares y procedimientos invasivos, lo que afecta la salud y el pronóstico de los pacientes.

**Conclusiones:**

Es fundamental considerar el sueño como un factor modificable en la prevención y manejo de la hipertensión en pacientes hospitalizados y encontrar intervenciones para mejorar su duración y calidad.

## Resumo

**Introdução:**

Vários estudos têm associado a duração e a qualidade do sono às alterações da pressão arterial na população geral, considerando-a um fator de risco associado, mas há pouca informação sobre pacientes hospitalizados, que tendem a apresentar distúrbios do sono.

**Objetivo:**

Examinar a relação entre duração subjetiva do sono, latência do sono e pressão arterial sistólica e diastólica em pacientes hospitalizados.

**Materiais e métodos:**

Estudo transversal de 381 adultos mexicanos hospitalizados em um hospital de segundo nível em Tampico, Tamaulipas, México. A duração e a latência do sono foram avaliadas por autorrelato utilizando o componente do Índice de Qualidade do Sono de Pittsburg. Foram obtidos dados clínicos e sociodemográficos. Foi aprovado pelo comitê de ética.

**Resultados:**

Nos modelos ajustados, foi encontrada associação significativa e negativa entre horas subjetivas de sono e pressão arterial diastólica (β= -0,59; IC 95%: -1,80 - -0,10). Foi observada relação significativa e positiva entre latência do sono e pressão arterial sistólica (β= 1,48; IC 95%: -0,49 – 2,47).

**Discussão:**

Os resultados são semelhantes aos de estudos anteriores. A hospitalização pode afetar o sono devido a ambientes desconhecidos, ruídos, horários irregulares e procedimentos invasivos, afetando a saúde e o prognóstico dos pacientes.

**Conclusões:**

É essencial considerar o sono como um fator modificável na prevenção e gestão da hipertensão em pacientes hospitalizados e encontrar intervenções para melhorar a sua duração e qualidade.

## Introduction

Arterial hypertension is one of the main modifiable risk factors for cardiovascular disease, chronic kidney disease, and some forms of dementia^1^-^8^. In 2019, the prevalence of hypertension worldwide was estimated to be 32% in women (95% CI: 30 – 34) and 33% in men (95% CI: 30 – 35), with higher prevalence in low-income and middle-income countries^9^. Several risk factors have been associated with changes in blood pressure; one of the most important is sleep. 

From a physiological point of view, blood pressure is known to have diurnal and nocturnal patterns related to the sleep-wake cycle; it is also influenced by endogenous circadian rhythms, as well as cyclic exogenous factors^10^. However, sleep is the most important source of circadian variation in blood pressure because each stage of sleep has an effect on blood pressure regulation^11^. Under normal circumstances, blood pressure drops to its lowest levels during nighttime sleep, reaching its lowest levels during stage N3 of non-REM sleep (without rapid eye movement), ranging from 10% to 20% compared to daytime blood pressure^12^. Thus, continual awakenings or any change in sleep quality or quantity can lead to fragmentation or interruption of cycles and, therefore, an increase in nocturnal blood pressure, which in turn influences the development of hypertension^13^,^14^. Furthermore, many physiological mechanisms that induce sleep or determine arousals play an important role in mediating the influences of sleep on blood pressure, including the diurnal predominance of the sympathetic nervous system and the nocturnal predominance of the parasympathetic nervous system^15^,^16^. Alteration of one or more of the mechanisms involved in sleep onset and arousals may also be reflected in altered circadian rhythms of blood pressure. 

In recent decades, numerous epidemiological studies have investigated how sleep duration and quality are related to high blood pressure^17^-^24^. A meta-analysis of 11 studies with 85,838 participants found a significant association between short sleep duration and hypertension (RR=1.61; 95% CI: 1.058 to 1.274)^25^. Other meta-analyses have found that both short and long sleep durations are associated with an increased risk of hypertension^17^,^26^,^27^. 

Several studies have reported similar results regarding sleep quality, such as the meta-analysis by Lo et al., which included 22 studies and reported that poor sleep quality is significantly associated with an increased risk of hypertension (OR=1.48, p-value=0.01)^22^. These associations may be due to various pathophysiological mechanisms, including sympathetic nervous system activation, endothelial dysfunction, disturbance of circadian rhythm, and systemic inflammation^13^,^22^,^28^. 

Additionally, recent research has highlighted the importance of considering not only sleep duration but also sleep latency—the time it takes to fall asleep after going to bed. It has been suggested that increased sleep latency may also be associated with an increased risk of elevated blood pressure^13^,^24^,^29^. 

Despite abundant evidence of this relationship in the general population, studies examining the association between sleep and blood pressure in hospitalized patients are scarce^30^. Hospitalization can cause significant disruptions in sleep patterns due to factors such as environmental noise, pain, emotional stress, and invasive medical procedures^31^-^33^, which can affect blood pressure levels. 

In this regard, a 2011 study conducted in the United States analyzed how sleep duration and quality were related to blood pressure in 20 hospitalized older adults^30^. It was reported that for each additional hour of sleep a patient lost, diastolic blood pressure increased by 6.2 mmHg (95% CI: 3.2 to 9.2); for systolic blood pressure, each 10% decrease in habitual sleep produced an increase of 4.2 mmHg (95% CI: 1.6 to 6.9)^34^. 

In Mexico, only one study has analyzed changes in the duration and quality of sleep among hospitalized patients; it reported that patients had a low amount of sleep, an average of 5 hours and 50 minutes, and 67.3% reported having poor sleep quality during hospitalization^34^. No studies have analyzed how sleep duration and quality affect blood pressure during hospitalization in Mexico. 

It is critical to analyze sleep latency and sleep duration in studies on blood pressure as both are directly related to cardiovascular regulation. Sleep duration influences the body's recovery processes, affecting mechanisms that regulate blood pressure^17^,^25^,^26^. Both too little and too much sleep is associated with an increased risk of hypertension^17^,^25^,^26^. Sleep latency, or the time it takes to fall asleep, is linked to stress and nervous system activation, factors that also affect blood pressure^35^. By focusing on these two variables, we avoid including potential confounding factors, such as daytime dysfunction or the use of sleep medications. 

Therefore, the main objective of this study was to determine how subjective sleep duration and sleep latency are related to systolic and diastolic blood pressure in hospitalized patients in Mexico. As secondary objectives, we sought to describe sleep duration, sleep latency, and sleep disturbances in these patients and to analyze whether the variables of age, sex, and sleep disturbances themselves could act as confounders in this relationship. Understanding these associations could have important clinical implications for blood pressure management and the overall well-being of hospitalized patients, as well as informing interventions to promote healthy sleep during hospitalization. 

## Materials and Methods

**Study design and participants**

A cross-sectional study was conducted in a Mexican population of adults hospitalized for at least one night in the Internal Medicine Unit at Dr. Carlos Canseco Hospital in Tampico, Tamaulipas. Participants were recruited from February to September 2022 through non-probabilistic convenience sampling, due to COVID-19 pandemic restrictions. The final sample included 381 patients who met the inclusion criteria and signed the informed consent form. 

**Inclusion and exclusion criteria**

The inclusion criteria required participants to be adults from the Mexican population who had been hospitalized for at least one night in the Internal Medicine Unit at Dr. Carlos Canseco Hospital in Tampico, Tamaulipas. Patients were asked to present their Mexican voter identification card to verify their identity. Patient recruitment took place between February and September 2022. In addition, patients were required to sign an informed consent form to participate in the study. 

Pregnant patients, trauma patients, postoperative patients, patients with mental deficiencies, patients treated with sedatives, critically ill patients, patients in isolation, patients on peritoneal dialysis, or patients on mechanical ventilators were excluded because these circumstances may interfere with normal sleep patterns. Participants with extreme systolic and diastolic blood pressure values were also excluded (n=22). 

**Blood pressure measurement and covariates**

Previously trained personnel conducted face-to-face interviews with patients who agreed to participate in the study. Interviews were conducted 24 hours after hospitalization to obtain basic sociodemographic information, including age, sex, occupation, education, and hospitalization data. Information was collected on self-reported chronic conditions such as previous diagnoses of diabetes mellitus, hypertension or other chronic disease. In addition, the presence of sleep disturbances during hospitalization, as measured by component 5 of the Pittsburgh Sleep Quality Index (PSQI), was considered. PSQI component 5 considers the following factors that disturb sleep: waking in the middle of the night, getting up to go to the bathroom, not being able to breathe comfortably, coughing or snoring loudly, feeling too cold, feeling too hot, having nightmares, and having pain. The component score is obtained by the patient's self-reporting of the frequency with which they experience sleep problems due to these factors (from not once to three or more times). Then, the total score is obtained, which ranges from 0 to 27 points, where the higher the score, the greater the frequency of sleep disturbances. The value obtained in this component was named “sleep disturbances.” After the first night of hospitalization, blood pressure was measured in the morning between 7:00 and 8:30 using a stethoscope and a newly calibrated manual aneroid sphygmomanometer. The patient was positioned in the semi-Fowler’s position, the brachial pulse was confirmed, and the sphygmomanometer cuff was placed 3 to 4 cm above the arm’s flexion line. It was then gradually inflated to a pressure between 180 and 200 mmHg, while systole and diastole sounds were auscultated. The procedure was repeated on both arms, and the average of the measurements was calculated and recorded in the demographic data form. Normal values for systolic pressure were 120 to 129 mmHg; for diastolic pressure, a value of 80 to 84 mmHg was considered normal (NOM-030-SSA2-1999)^36^. 

**Sleep measurement**

The main exposure variable was sleep, which was measured through two variables: subjective sleep hours and sleep latency. In both cases, information was obtained during the interview using the Pittsburgh Sleep Quality Index (PSQI) measures. The PSQI is a widely recognized tool for assessing sleep in adults. This self-assessment questionnaire consists of 19 questions designed to address various aspects of sleep, such as duration, efficiency, time taken to fall asleep, subjective sleep quality, and possible disorders^37^. Developed by Buysse et al. in 1989, the instrument has been translated into several languages, including Spanish, and has been validated in various Spanish-speaking populations, such as Spain and Colombia^37^-^39^, as well as in specific groups, such as the Mexican psychiatric population^40^. Validation in Spain demonstrated high reliability, with a Cronbach's alpha coefficient of 0.81, and showed a sensitivity of over 88% and a specificity of 75%^39^. 

Regarding the subjective sleep hours variable, the question was: *“During your hospitalization, how many hours did you feel you slept?* Sleep latency hours were assessed using the PSQI component 2, which consists of two questions: *(1) During your hospitalization, how long did it take you to fall asleep? and (2) During your hospitalization, how many times did you have trouble sleeping because you could not get to sleep within the first half hour?*(1) During your hospitalization, how long did it take you to fall asleep? and (2) During your hospitalization, how many times did you have trouble sleeping because you could not get to sleep within the first half hour? 

The sleep latency variable was obtained by summing the response scores, which give values between 0 and 6; the higher the score, the longer it took the participant to fall asleep, and the greater the weekly frequency of not falling asleep within the first half hour. The details of how these scores were obtained are shown in Appendix 1. 

** Statistical analysis**

Descriptive statistics were calculated for participants' main sociodemographic variables. For the categorical variables, percentages and frequencies were calculated; for the numerical variables of age and systolic and diastolic blood pressure, means with standard deviation (SD) were calculated; and for the variables of days of hospitalization and sleep disturbance score, medians and interquartile ranges (Q1-Q3) were calculated, since these variables did not meet the assumption of normality (Shapiro-Francia test, p<0.05). 

Subsequently, the relationship between the included variables and subjective sleep hours and sleep latency was analyzed. For numerical variables, Spearman correlation coefficients were calculated (age, days of hospitalization, systolic and diastolic blood pressure, and sleep disturbance score); for categorical variables, the Mann Whitney U test (sex, living arrangement, diabetes, hypertension, other chronic disease), Kruskal Wallis test (education) or ANOVA (occupation) were used. 

Finally, to estimate the associations between subjective sleep hours and sleep latency with systolic and diastolic blood pressure, both crude and adjusted linear regression models were run, after assessing the normality of the outcome variables (Shapiro-Francia test, p> 0.05). Subjective sleep hours and sleep latency were each considered independently as explanatory variables and systolic and diastolic blood pressure were each treated independently as outcome variables, resulting in four regression models. The adjustment variables were age, sex, self-reported medical diagnosis of diabetes, hypertension, or other chronic diseases, as well as sleep disturbances because they were considered potential confounding variables. The adjusted models met the assumptions of linearity (E[ei]≈ 0), normality (Shapiro-Francia test, p> 0.05), homoscedasticity (Breusch-Pagan / Cook-Weisberg test, p> 0.05), independence (Durbin-Watson ≈ 2 statistic) and absence of multicollinearity (VIF< 10). Stata 14.0 statistical software for Windows (StataCorp LLC, College Station, TX, USA) was used for all analyses. The database with the results obtained is available in the Mendeley Data repository^41^.

**Ethical considerations**

The study was deemed "risk-free" according to ethical standards, as it involved no direct intervention or manipulation of patients and focused solely on collecting information through interviews and questionnaires without obtaining biological samples or performing invasive procedures. Patients participated voluntarily after receiving a detailed explanation of the study and signing an informed consent form. Privacy and confidentiality were ensured in accordance with the principles of the Declaration of Helsinki. Additionally, approval was obtained from the Research Ethics Committee of Dr. Carlos Canseco Hospital on January 27, 2022, for the original project, “Sleep and Vital Signs in Hospitalized Patients,” under registration number 001/2022/CEI-HGT. 

## Results

The sample consisted of a total of n=381 participants. The mean age was 56.96 years (SD = 14.98), and the majority of participants were male (54.59 %) (Table 1). In total, 56.96% of the participants live with someone, 34.38% are employed, and 40.94% have elementary school or no education. The prevalence of diabetes was 54.07%, while the prevalence of hypertension was 60.10%; 15.49% reported having another chronic disease. The median number of days of hospitalization was 4 days (Q1-Q3= 3 – 7 days), the mean systolic blood pressure was 123.79 mmHg (SD= 15.85 mmHg), and the mean diastolic blood pressure was 76.69 mmHg (SD= 11.30 mmHg). 

Regarding sleep variables, the median subjective sleep hours reported by participants was 5 hours (Q1-Q3= 4 – 6 hours), the median total score of PSQI component 2 measuring sleep latency was 3 points (Q1-Q3= 1 – 4 points), and the median sleep disturbance score was 8 points (Q1-Q3= 5 – 12 points). 

**Table 1:** Sociodemographic and health characteristics by sleep quality components

Categorical variables	Total n (%)	Subjective sleep hours~	p-value	Sleep latency~	p-value
A. Categorical variables					
Sex			0.90		0.70
Man	208 (54.59)	5 (4 – 6)		2.5 (1 – 4)	
Woman	173 (45.41)	5 (3.3 – 7)		3 (1 – 4)	
Living arrangement			0.47		0.77
Living with others	217 (56.96)	5 (4 – 6)		3 (1 – 4)	
Living alone	164 (43.04)	5 (3 – 6,5)		3 (1 – 4)	
Occupation*			0.67		0.61
Homemaker	119 (31.23)	5 (4 –7)		2.51 (1.71)	
Employee	131 (34.38)	5 (4 – 7)		2.72 (1.75)	
Pensioner/ Retiree	40 (10.50)	5 (3.65 – 6)		2.35 (1.76)	
Other	91 (23.88)	5 (3 – 6)		2.58 (1.66)	
Education			0.63		0.36
Elementary or none	156 (40.94)	5 (3 – 6)		2 (1 – 4)	
Middle School	95 (24.93)	5 (4 – 7)		3 (2 – 4)	
High School	74 (19.42)	5 (4 – 7)		3 (0 – 4)	
University	56 (14.70)	5 (3 – 6)		3 (1 – 4.5)	
Diabetes			0.24		0.11
No	175 (45.93)	5 (3 – 6)		3 (1 – 4)	
Yes	206 (54.07)	5 (4 – 7)		2 (1 – 4)	
Hypertension*			0.01		0.04
No	152 (39.90)	5.42 (2.25)		2.79 (1.70)	
Yes	229 (60.10)	4.94 (2.27)		2.44 (1.72)	
Other chronic disease			0.59		0.70
No	322 (84.51)	5 (4 – 6)		3 (1 – 4)	
Yes	59 (15.49)	4 (4 – 6)		3 (1 – 4)	
B. Numerical variables					
Age, years	56.96 ± 14.59	-0.01	0.76	-0.16	<0.001
Days of hospitalization^	4 (3 – 7)	0.08	0.11	0.18	<0.001
Systolic blood pressure	123.79 ± 15.85	-0.14	0.01	0.13	0.01
Diastolic blood pressure	76.69 ± 11.30	-0.18	<0.001	0.08	0.12
Sleep disturbance score^°	8 (5 – 12)	-0.27	<0.001	0.47	<0.001

n= 381. + n(%) is presented for categorical variables and mean and SD for numerical variables, except for ^, where the median is reported (Q1–Q3). ~ For categorical variables, median and (Q1–Q3) are presented unless otherwise indicated. For numerical variables, Spearman's correlation coefficient is displayed. ‘ The p-value presented corresponds to the difference between the reported means or medians (Mann-Whitney U test, Kruskal-Wallis, ANOVA) for categorical variables and to the correlation coefficient for numerical variables. *Mean (SD) is reported. ° Sleep disturbances were measured with the PSQI^ component 5 score.

Table 1 compares sociodemographic and health characteristics with components of sleep quality. It can be seen in the first section of this table that, of the categorical variables evaluated, a significant difference was found in the hypertension variable and the sleep disturbance score, where the group that reported having a diagnosis of hypertension had a lower average number of sleep hours (4.9 hours vs 5.3 hours, p= 0.028) and a lower sleep latency (2.44 points vs 2.79 points, p= 0.04) compared to those who did not have a diagnosis. Regarding sleep disturbances, a negative correlation was observed with subjective sleep hours (-0.27, p<0.001) and a positive correlation with sleep latency (0.47, p<0.001). 

Table 1, in the continuous variables section B, shows Pearson's correlation coefficients with subjective sleep hours and sleep latency. Subjective sleep hours were negatively and significantly correlated with systolic blood pressure (ρ= -0.14; p= 0.01) and diastolic blood pressure (ρ= -0.18; p< 0.001). Sleep latency was negatively correlated with age (ρ= -0.16; p<0.001) and positively and significantly correlated with days of hospitalization (ρ= 0.18; p<0.001) and systolic blood pressure (ρ= 0.13; p= 0.01). 

Table 2 shows the results of the crude and adjusted linear regression models of subjective sleep hours and sleep latency with systolic and diastolic blood pressure. In crude models, subjective sleep hours were negatively associated with both systolic blood pressure (β= -1.01; 95% CI: -1.70 – 0.32) and diastolic blood pressure (β= -0.91; 95% CI: -1.40 – -0.41), indicating that the more hours of sleep, the lower the blood pressure. Regarding sleep latency, a positive relationship was also found with systolic blood pressure (β= 1.38; 95% CI: 0.46 – 2.30) and diastolic blood pressure (β= 0.65; 95% CI: -0.01 – 1.31); this result indicates that the longer the time to fall asleep, the more both diastolic and systolic blood pressure will increase. In models adjusted for age, sex, medical self-report of diabetes, hypertension, or other chronic disease, as well as sleep disturbance score, the association of subjective sleep hours with diastolic blood pressure and sleep latency with systolic blood pressure were maintained. However, a marginal negative association of subjective sleep hours with systolic blood pressure was observed (p= 0.10), while the coefficient of the association of sleep latency with diastolic blood pressure lost statistical significance (p= 0.39). 

**Table 2:** Crude and adjusted linear regression models of systolic and diastolic blood pressure by sleep quality components

Sleep quality components	Systolic blood pressure			Diastolic blood pressure		
	β	95% CI	p-value	β	95% CI	p-value
Crude models						
Subjective sleep hours	-1.01	(-1.70 – -0.32)	<0.01	-0.91	(-1.40 – -0.41)	<0.001
Sleep latency, total component score	1.38	(0.46 – 2.30)	<0.01	0.65	(-0.01 – 1.31)	0.05
Adjusted models*						
Subjective sleep hours	-0.56	(-1.24 – 0.12)	0.10	-0.59	(-1.08 – -0.10)	0.02
Sleep latency, total component score	1.48	(0.49 – 2.47)	<0.01	0.31	(-0.41 – 1.03)	0.39

*Adjustment variables: age, sex, diabetes, hypertension, other chronic diseases and sleep disturbances n=381

## Discussion

This study found a significant inverse association between subjective sleep hours and systolic and diastolic blood pressure in hospitalized patients. In addition, sleep latency in these patients was also positively and significantly associated with both diastolic and systolic blood pressure.

The available scientific evidence consistently shows how sleep duration affects blood pressure and increases the risk of hypertension^17^,^25^,^26^; however, evidence in hospitalized populations is scarce. A previous study showing an association between sleep duration and hypertension in 20 hospitalized older adults found that each additional hour of sleep loss was associated with an increase in blood pressure of 6.2 mmHg (95% CI: 3.2 – 9.2) for diastolic pressure and 3.1 mmHg (95% CI: 1.6 – 4.6) for systolic pressure^30^. Although the results are consistent with those of this study regarding the importance of sleep duration, there are discrepancies in the magnitude of the coefficients; in the results of the adjusted model, the change in blood pressure was -0.59 (95% CI: -1.08 – -0.10) for diastolic pressure and -0.56 (95% CI: -1.24 – 0.12) for systolic pressure. This may be related to differences in the models, the intervening variables included, and the operationalization of variables. Additionally, there are differences in the study populations, particularly regarding participants' age and sleep hours during hospitalization. 

The study did not estimate the sleep hours lost during hospitalization, but if we consider that the average reported hours of sleep in the Mexican adult population is 7 hours 19 minutes^42^ and compare it with the 5 hours and 50 minutes reported by the participants, we can estimate a decrease of 1 hour and 29 minutes of sleep. This is a reduction in sleep less than that reported by Arora et al., which was 2.5 hours of habitual sleep^30^. However, it would be necessary to confirm the average sleep duration with the participants under non-hospital conditions. These findings support the idea that sleep disturbance may be an important risk factor for arterial hypertension in various groups of hospitalized patients. 

When the results of this study are compared with those of the non-hospitalized population, there are similarities. In the case of the CARDIA sleep study, for example, a relationship was found between sleep hours and blood pressure; the authors reported a decrease of 1.80 mmHg (95% CI: -3.07 – -0.52) in systolic blood pressure for each additional hour of sleep; in the case of diastolic blood pressure, the effect was 1.70 mmHg (95% CI: -2.72 – -0.68)^21^. The magnitude of the coefficients in this study was lower, which may be attributed not only to population differences but also to the precision of the measurement instruments since the CARDIA study used wrist actigraphy for three consecutive days to assess sleep duration and, in our case, self-reporting was used, which may lead to lower precision in the measurement of the variable. 

Regarding sleep latency, no studies in hospitalized patients have reported an association. However, our results are consistent with previous research in a non-hospitalized population that found that a longer sleep latency was associated with higher blood pressure. However, our results are consistent with previous research in a non-hospitalized population that found that a longer sleep latency was associated with higher blood pressure^13^,^23^,^24^. Despite the differences between the hospital and the general populations, the results are very similar. The study by Zhong et al. reported positive correlations between sleep latency and systolic blood pressure (r = 0.186; p < 0.001) and diastolic blood pressure (r = 0.136; p < 0.001), values very similar to those obtained in our study, with a ρ = 0.13 (p = 0.01) for systolic blood pressure and ρ = 0.08 (p = 0.12) for diastolic blood pressure^24^. 

Hospitalization can cause a series of sleep disturbances in patients^31^. Exposure to unfamiliar and noisy environments, irregular medication schedules, invasive medical procedures, and conditions associated with the illness or procedure that led to hospitalization can all contribute to disruptions in the natural sleep cycle^31^,^43^. As a result, many hospitalized patients experience a significant decrease in total sleep duration. In this regard, Wesselius et al. found an average decrease of 83 minutes with respect to habitual sleep; in addition, they also reported a decrease in sleep quality^31^. Venkateshiah et al. highlighted the frequency of sleep abnormalities in hospitalized patients, including reduced total sleep time and increased sleep fragmentation, as well as the possible role of environmental noise and circadian rhythm disruptions^44^. These sleep disturbances can have negative consequences on the health of patients and on the prognosis of the diseases or procedures for which they are hospitalized. 

The variable of sleep disturbance, which has been shown to be more prevalent and severe in the hospital population, was included in this study^44^. This variable is important because it is usually not included in many studies that analyze the relationship between sleep and blood pressure. The results obtained showed a negative correlation with subjective sleep hours (-0.27, p<0.001) and a positive correlation with sleep latency (0.47, p<0.001). This finding indicates that the greater the number of disturbances, the shorter the sleep duration and the greater the difficulty in falling asleep. These results underscore the importance of considering sleep disturbances when analyzing the relationship between sleep and blood pressure in a hospital setting. 

This suggests that studies with higher coefficients may be affected by residual confounding, as they often do not include variables such as sleep disturbances, potentially biasing the true association between sleep and blood pressure. By accounting for this variable, our study reduces the possibility of residual confounding and provides a more accurate estimate of the relationship between sleep and blood pressure. 

In this sense, it is essential to recognize sleep disturbances as part of the hospital care process and to take steps to mitigate their impact on patient health. Strategies like promoting a calmer hospital environment and implementing sleep hygiene programs can help enhance the quality and duration of patients' sleep during hospitalization^31^. 

Finally, it is important consider some limitations of this study. First, the cross-sectional nature of the design limits the ability to establish causal relationships between variables. Additionally, information on sleep duration and quality relied on patient self-report, which may be subject to bias and recall errors. Future research would benefit from longitudinal studies to better understand patients' sleep dynamics before hospitalization. It would also be advisable to use objective sleep measurement methods, such as polysomnography, to obtain a more accurate assessment of sleep quality and its relationship to blood pressure. 

## Conclusions

This study identified a significant inverse association between subjectively reported sleep hours and diastolic blood pressure in hospitalized patients. The longer the sleep duration, the lower the blood pressure, which is consistent with previous literature. In addition, a positive association was found between sleep latency and systolic blood pressure, suggesting that difficulty falling asleep is associated with higher blood pressure levels. The results remained significant when adjusting for confounding variables such as sex, age, sleep disturbances, diabetes, hypertension, and other chronic diseases. The sleep disturbance variable was significantly correlated with both sleep duration and sleep latency and was significant as a confounder in the adjusted models. These results underscore the need to account for sleep disturbances in future studies, as their omission could cause residual confounding and bias the true association between sleep and blood pressure. 

The findings underscore the importance of considering sleep as a modifiable risk factor in preventing and managing blood pressure in hospitalized patients. Early identification of sleep problems and implementation of interventions aimed at improving sleep quality and duration in hospitalized patients may significantly benefit cardiovascular health.
